# 4-Thiazolidinone Derivatives as MMP Inhibitors in Tissue Damage: Synthesis, Biological Evaluation and Docking Studies

**DOI:** 10.3390/molecules23020415

**Published:** 2018-02-14

**Authors:** Matteo Incerti, Lucia Crascì, Paola Vicini, Esin Aki, Ismail Yalcin, Tugba Ertan-Bolelli, Venera Cardile, Adriana Carol Eleonora Graziano, Annamaria Panico

**Affiliations:** 1 Department of Food and Drug, University of Parma, Parco Area delle Scienze 27/A, 43124 Parma, Italy; matteo.incerti@unipr.it (M.I.); paola.vicini@unipr.it (P.V.); 2 Department of Drug Sciences, University of Catania, V. le A. Doria 6, 95125 Catania, Italy; panico@unict.it; 3 Pharmaceutical Chemistry Department, Faculty of Pharmacy, Ankara University, Tandogan, Ankara 06100, Turkey; esinaki@ankara.edu.tr (E.A.); yalcin@ankara.edu.tr (I.Y.); tbolelli@ankara.edu.tr (T.E.-B.); 4 Department of Physiological Sciences, University of Catania, V. le A. Doria 6, 95125 Catania, Italy; cardile@unict.it (V.C.); acegraziano@tiscali.it (A.C.E.G.)

**Keywords:** 4-thiazolidinones, ORAC assay, metalloproteinase-9, docking study, keratinocytes cultures, nuclear factor-κB

## Abstract

Nine 2-(1,2-benzothiazol-3-yl)-*N*-(4-oxo-2-phenyl-1,3-thiazolidin-3-yl)propanamides combining a benzisothiazole and 4-thiazolidinone in one framework were designed and synthesized. The aim of the study was to verify their effectiveness to affect the inflammatory/oxidative process in which free oxygen and nitrite (ROS and RNS) radicals, inflammatory mediators, such as nuclear factor κB (NF-κB), and matrix metalloproteinases (MMPs) are involved. Docking studies of all the compounds were performed in order to explore their binding mode at the MMP-9 protein. An appreciable anti-inflammatory/potential wound healing effects of the tested compounds was highlighted. Derivative **23**, bearing a 4-carboxyphenyl substituent at C2 of the 4-thiazolidinone ring, exhibited the highest activity, being able to inhibit MMP-9 at nanomolar level(IC_50_ = 40 nM).

## 1. Introduction

**Figure 1:**
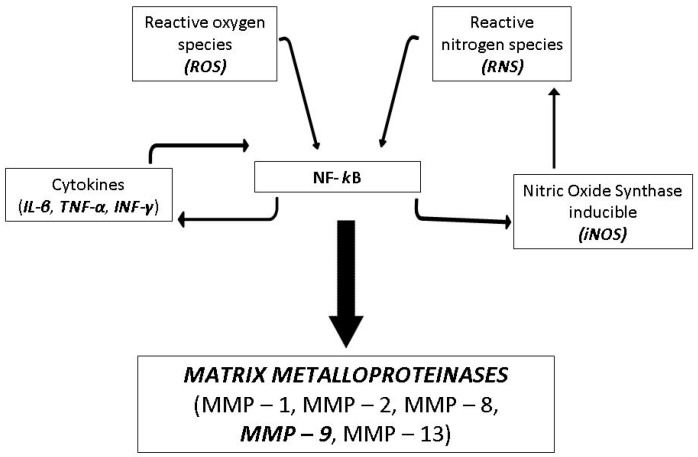
MMPs activation via oxygen and nitrite (ROS and RNS) radicals, pro-inflammatory cytokines and growth factors) and inducible nitric oxide synthase (iNOS).

Chronic inflammation causes tissue damage and has been identified as contributing to a host of diseases, including cardiovascular, pulmonary, neurodegenerative, metabolic/obesity-related diseases [1]. The inflammatory event is activated by free oxygen and nitrite (ROS and RNS) radicals, advanced glycation products (AGEs), inflammatory cytokines and matrix metalloproteinases (MMPs) that act repeatedly and inappropriately. Moreover, it is well established that accumulation of ROS and advance glycation products (AGEs), can activate a cascade of radical reactions, causing the up-regulation of MMPs production, via activation of eukaryotic nuclear factor κB (NF-κB) pathway [2]. NF-κB is a transcription factor that is a regulator of immune responses stimulated by pro-inflammatory agents such as interleukin-1β (IL-1β) and tumor necrosis factor α (TNF-α), and is sensitive to cellular redox modifications [3]. ROS appears to be responsible for IL-1β-induced NF-κB activation and inducible nitric oxide synthase (iNOS) expression [4]. RNS, as nitric oxide (NO) and peroxynitrite, in turn, exert proinflammatory and catabolic effects, by providing a persistent activation of NF-κB and, consequently, NF-κB-dependent transcription of MMPs (Figure 1) [2].

An increased level of MMPs, as elastase, plasmin and thrombin, destroy components of the extracellular matrix (ECM), causing chronic inflammation and tissue damage. Tissue integrity is a complex biological process that involves ECM remodeling. The ECM is composed of a variety of polysaccharides, water and collagen proteins which give the skin remarkable properties. In tissue damage of the vascular endothelium high levels of MMPs destroy ECM components.In particular, epithelial-derived MMP-9 is an important mediator of tissue injury [5].

The MMPs are part of a large family of enzymatic calcium-dependent endopeptidase containing zinc. They are produced mainly by connective tissue cells and are responsible of tissue remodeling and degradation of the ECM components (collagen, elastin, gelatin, matrix of glycoproteins and peptidoglycans), through the hydrolysis of peptide bonds at the level of specific amino acid sequences.

The use of specific and selective MMPs inhibitors may lead to monitoring and improvement of pathologies state. Most of MMPs maintains a common structural features, such as the presence of a catalytic zinc ion and many equalities in the amino acid sequences in the binding site [6]. MMPs inhibitor possess a functional group able to chelate the catalytic zinc present in the active site of the enzyme, the Zinc-Binding-Group (ZBG), and a planar and lipophilic portion able to mainly occupy the binding site S1′. Derivatives with hydroxamic function result potent and effective inhibitors [7]. However, they show toxicity and poor bioavailability prompting the search for new molecules with greater efficacy and selectivity.

In last decade our group conduced an intensive research program focused on the design and synthesis of new chondroprotective/anti-inflammatory molecules, our interest in this field prompted us to study the thiazolidinones, that have been demonstrated to possess anti-inflammatory properties and a protective action on inflammatory degenerative diseases such as osteoarthritis (OA) [8,9,10,11]. The new class of benzo[*d*]isothiazolyimino-5-benzylidene derivatives showed an interesting activity in inhibition of MMP-3 and MMP-13 by a model that reproduces the mechanism involved in OA diseases [12,13]. 

From the above considerations in the present work we decided to investigate a novel class of 4-thiazolidinone derivatives as MMPs inhibitors in tissue damage. In particular, considering the involvement of MMP-9 in tissue damage and its high expression levels in epithelial tissue [5] ROS production correlated, we assayed MMP-9 inhibitory effect (EC_50_) and antioxidant activity (ORAC assay).

Thus, nine2-(1,2-benzothiazol-3-yl)-*N*-(4-oxo-2-phenyl-1,3-thiazolidin-3-yl)propanamides were designed and synthesized. The designed compounds combine two bioactive moieties—benzisothiazole and 4-thiazolidinone—in one skeleton. We planned to introduce also an isopropanoylhydrazide spacer, between the two heterocycles, aimed at emphasizing their antinflammatory/protective effects on keratinocytes. All of the novel 4-thiazolidinone derivates bear an aromatic moiety at C2 of the 4-thiazolidinone ring with hydrophobic/hydrophilic/hydrogen-donor group at C4 to evaluate their importance for activity. Furthermore, docking studies of all of the compounds were performed in order to explore the binding mode at the MMP-9 protein, helping us to gain understanding of the pharmacophoric requirements necessary for the activity and for the rational design of new inhibitors.

## 2. Results and Discussion

### 2.1. Synthesis

**Scheme 1:**
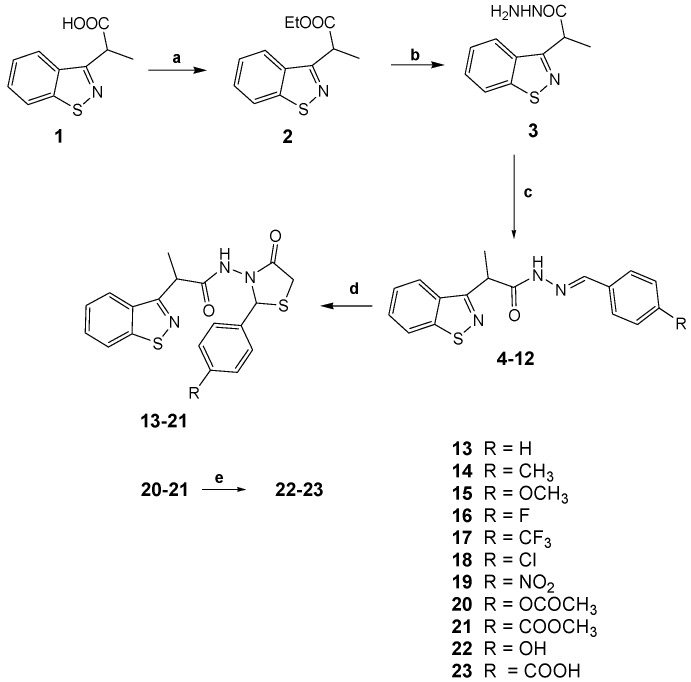
General procedure for the synthesis of compounds **1**–**23**. General procedure for the synthesis of compounds **1**–**23**. *Reagents and conditions*: (a) conc. H_2_SO_4_, EtOH, reflux, 8 h; (b) NH_2_NH_2_, MeOH, r.t., 2 h; (c) R-C_6_H_4_CHO, CH_3_COOH, EtOH, reflux, 8 h or MFB or FPA, CH_3_COOH, EtOH, reflux, 5 h; (d) HSCH_2_COOH, CH_3_C_6_H_5_, reflux, 48 h; (e) NaOH, EtOH, r.t., 2 h; then HCl.

The target compounds **13**–**19**, **22** and **23** were synthesized using a stepwise reaction protocol (Scheme 1), starting from 2-(1,2-benzothiazol-3-yl)propanoic acid (**1**), synthesized starting from 3-chloro-1,2-benzothiazole using procedures reported earlier [14]. Heating **1** and ethanol in the presence of concentrated sulfuric acid gave 2-(1,2-benzothiazol-3-yl)propanoic acid ethyl ester (**2**). By reacting this ethyl ester and hydrazinehydrate in methanol 2-(1,2-benzothiazol-3-yl)propanoic acid hydrazide (**3**) was obtained [15]. Hydrazide **3** was allowed to react with commercially available aldehydes or with 4-formylphenyl acetate (FPA) [16] and methyl 4-formylbenzoate (MFB) [17], obtained in turn through reactions starting from commercially available aldehydes. FPA and MFB were prepared to convert the hydroxyl and carboxyl group of 4-hydroxy- and 4-carboxybenzaldehyde in their ester derivatives with the purpose to avoid decomposition that occurred in the final cyclocondesation step without such protection. Refluxing an ethanolic solution of **3** with the suitable aldehydes in the presence of acetic acid led to 2-(1,2-benzothiazol-3-yl)-*N*′-phenylmethylidenepropanehydrazides **4**–**12**. Reaction (cyclocondesation) of the hydrazides **4**–**12** with sulfanylacetic acid in refluxing toluene, under a N_2_ atmosphere, afforded 2-(1,2-benzothiazol-3-yl)-*N*-(4-oxo-2-phenyl-1,3-thiazolidin-3-yl)propanamides **13**–**21** in good yield. Finally, deprotection of **20** and **21** under basic conditions, led to the formation of the target derivatives **22** and **23**.

### 2.2. Biological Evaluation

#### 2.2.1. Antioxidant Capacity

Free radicals and ROS are highly reactive molecules that are generated by normal cellular processes. They react with cellular components, damaging DNA, carbohydrates, proteins, and lipids causing cellular and tissue injury. Although organisms have developed complex antioxidant systems to protect themselves from oxidative substances, an excess of ROS production can lead to inflammation and several disease state. ORAC test measures both lipophilic and hydrophilic antioxidant capacity and determines the ability of antioxidants to protect proteins from damage caused by free radicals [18]. ORAC assay has been widely accepted as a standard in vitro to measure the antioxidant activity of synthetic compounds, measuring their capacity to transfer hydrogen atom. The assay measures the loss of fluorescence over time due to peroxyl-radical formation by the breakdown of 2,2′-azobis-2-methyl-propanimidamide dihydrochloride (AAPH). 6-Hydroxy-2,5,7,8-tetramethylchroman-2-carboxylic acid (Trolox), a water soluble vitamin E analog, is a positive control that inhibits the fluorescein decay in a dose dependent manner. The ORAC assay is a kinetic assay measuring fluorescein decay and antioxidant protection over time. 

The tested 2-(1,2-benzothiazol-3-yl)-*N*-(4-oxo-2-phenyl-1,3-thiazolidin-3-yl)propanamides **13**–**19**, **22** and the analogue **23** exhibited antioxidant activity, being able to delay the reduction of fluorescence of a fluorescein-AAPH solution appreciably (Table 1). In particular, compound **22** bearing a 4-hydroxyphenyl substituent at C2 of the 4-thiazolidinone ring showed excellent antioxidant effectiveness, producing appreciable effect than the reference Trolox (Table 1); the presence of a hydrophilic, electron donating and hydrogen bond forming group (OH) determines a greater activity than the analogues.

#### 2.2.2. Inhibitory Activity on MMP-9 

MMPs play crucial roles in many physiological or pathological events including tissue remodeling, ECM breakdown and the processing of a variety of biological molecules. MMP-9 is a Zn^+2^ dependent endopeptidase, synthesized and secreted in monomeric form as zymogen. Its primary function is degradation of proteins and particularly digests gelatin (denatured collagen), and types IV, V, XI and XVI collagen. Physiologically, MMP-9 in coordination with other MMPs, play a role in normal tissue remodeling events such as wound healing [19]. 

The synthesized compounds **13**–**19**, **22** and **23** were tested for their in vitro ability to inhibit human recombinant MMP-9. Table 2 reports the results of this inhibition assay, expressed as IC_50_ (µM). Most of the tested compounds inhibited MMP-9, with IC_50_ values in the low micromolar range. We used as positive control a potent MMPs inhibitor, NNGH (*N*-isobutyl-*N*-(4-methoxy-phenylsulfonyl)glycylhydroxamic acid) [20]. Compounds **22** and **23**, bearing hydrophilic and hydrogen bonds forming groups (OH and COOH), were the most active derivatives, with a IC_50_ value of 0.30 µM and 0.04 µM, respectively. Conversely the introduction, on the 4 position of the phenyl moiety at C-2 of the 4-thiazolidinone ring, of a less hydrophilic group (OCH_3_) or lipophilic methyl, nitro and halogens (Cl, F) substituents, led to 22 to 550 fold less potent MMP-9 inhibitors. Only the compound with the 4-trifluoromethylphenyl substituent at C-2 of the 4-thiazolidinone ring did not show any inhibitory effectiveness toward MMP-9.

#### 2.2.3. Cellular Assay

In vitro biological activity of compounds **13**–**19**, **22** and **23** was evaluated at different concentrations (100, 50 and 10 μM) using human keratinocytes (NCTC 2544) stimulated for 48 h with 200 U/mL of IFN-γ and 10^−4^ M of histamine (H). All tested compounds reduce the ability of NCTC 2544 to metabolise tetrazolium salts at the doses of 100 and 50 μM, but not at 10 μM, demonstrating in this case that they did not interfere with cell viability (data not shown).

#### 2.2.4. Determination of NF-κB Levels

Wound healing is a highly coordinated and complex process involving various cell types, chemical mediators and ECM components. The human keratinocytes treated with pro-inflammatory stimuli, as interferon gamma (IFN-γ) and histamine (H) represent a models of cutaneous wound healing. In fact, we demonstrate their potential as compounds useful in this process indirectly, through the reduction of NF-kappa B expression levels on keratinocytes cultures, in which an inflammatory process is triggered. 

**Figure 2:**
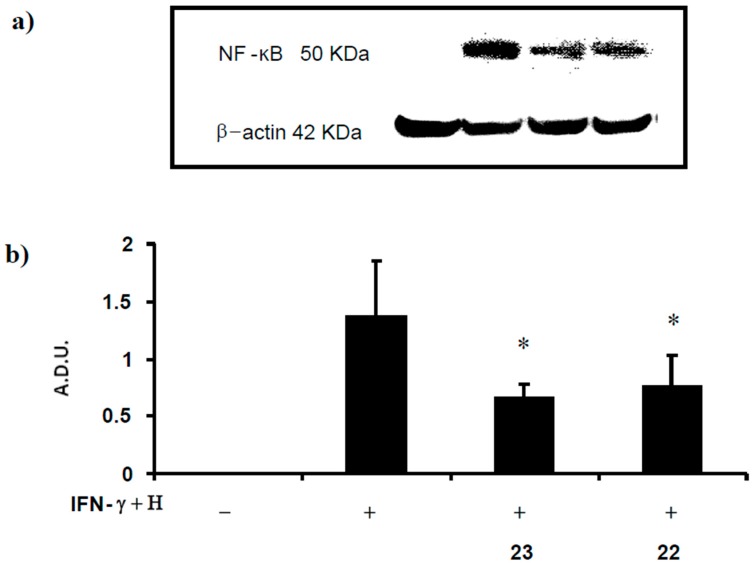
Effects of **22** and **23** on NF-κB expression. (**a**) Representative blot; (**b**) Nuclear Factor-kappa B (NF-κB) levels in cultures of human keratinocytes stimulated with IFN-γ and histamine (H), 48 h after the addition of compounds **22** and **23** at 10 µM concentrations, determined by Western blot analysis. Data show the relative expression (mean ± SEM) of NF-κB, calculated as arbitrary densitometric units (A.D.U.), from three independent experiments. * *p* < 0.05, compared with respective IFN-γ and H.

The inhibition of NF-κB expression, using western blot analysis, was evaluated on compounds **22** and **23**, the 4-thiazolidinones derivatives that possess the most potent inhibitory effect against MMP-9 and antioxidant activity (Figure 2).

The treatment of keratinocyte cell line NCTC 2544 with 200 U/mL of IFN-γ and 10^−4^ M of H increased NF-κB levels compared to untreated cells. IFN-γ is an essential cytokine in amplifying inflammatory reactions, as it stimulates the synthesis of chemokines that attract inflammatory cells and induces expression of molecules important for the retention and activation of T cells. We employed both IFN-γ and histamine because they use distinct signal transduction pathways, and this can lead to a stronger activation of inflammatory genes. Histamine is released from mast cells and keratinocytes in the early stage of inflammation of the skin and participates in the control of the inflammatory responses by acting on lymphocytes, monocytes, and leukocytes. Histamine binds to cell surface receptors coupling to guanine nucleotide-binding protein (G-protein) and induces various intracellular signalling pathways. These histamine-receptor-mediated signals regulate cytokine or chemokine gene expression in target cells [21]. Stimulated keratinocytes were treated with compounds **22** and **23** and the levels of NF-κB were measured after 48 h. All compounds reduced NF-κB release at 10 µM concentrations. The best results were obtained with **23** which provided up to 50% reduction of NF-κB levels compared to IFN-γ and H-treated cells.

The observed ability of compounds **22** and **23** to reduce NF-κB levels in keratinocytes appears to be of interest since this is an upstream event. In fact the NF-κB reduction can lead to decreased levels of other pro-inflammatory mediators, such as cytokines and different enzymes (such as MMPs, iNOS), interfering with downstream signaling components crucial for inflammatory response and degenerative processes involved in wound healing.

### 2.3. Docking Studies on MMP-9

To rationalize the observed activity data, all syntetized compounds were docked into the active site of MMP-9 using the software Discovery Studio 3.5 Accelrys Inc. San Diego, CA, USA, 2012 [22]. MMP inhibitors generally follow a two-component strategy, which is designed to interact in a noncovalent fashion with the MMP active site, while an appended zinc (II)-chelating moiety binds via coordinate–covalent bonds to the hydrolytic zinc (II) ion, rendering the enzyme inactive [23,24]. According to ZBG studies, using the coordinates of protein-ligand complex structures obtained from the PDB, the most frequently found ZBG is carboxylate, followed by sulfonamide, hydroxamate, and phosphonate/phosphate. Whereas carboxylates bound to the zinc via both monodentate and bidentate interactions, the hydroxamates bound dominantly in a bidentate manner [25]. The median distances from the nitrogen, oxygen, and sulfur atoms were 1.99, 2.05 and 2.28 Å respectively. The median distance from sulfur was the longest among these three heteroatoms. The differences among these distances were thought to be the result of the different sizes of the elements [26].

**Figure 3:**
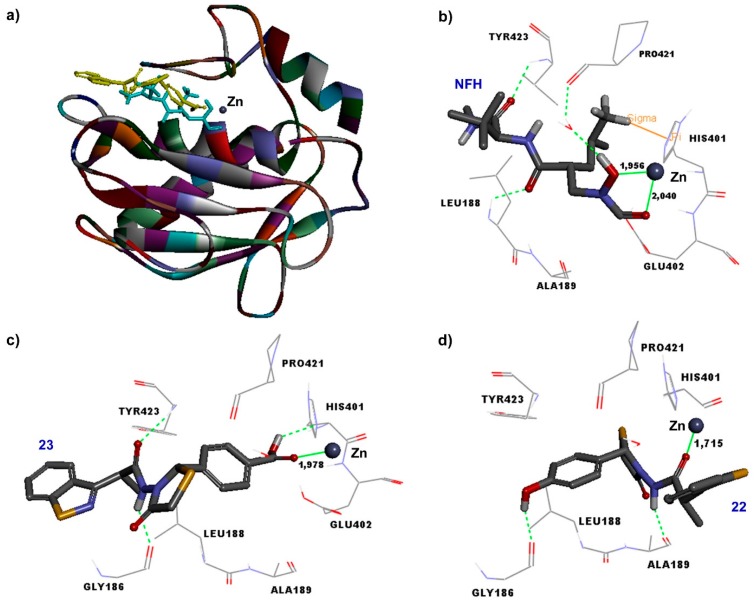
(**a**) Binding modes of **23** (yellow) and NFH (blue); (**b**) Docking position * of NFH: showed hydrogen bonds with Gly186, Leu188, Tyr421(water mediated), Tyr423 and a π–sigma interaction with His401; (**c**) Docking position * of **23**: showed hydrogen bonds with Gln186, Tyr423 and His401; (**d**) Docking position * of **22**: showed hydrogen bonds with Gln186, Ala189. * All hydrogen bonds are shown as green dashed lines and all distances are shown as green lines.

The catalytic centre of MMP-9 is composed of the active-site zinc ion, coordinated by three histidine residues (His401, His405 and His411) and the essential glutamic acid residue (Glu402). The crystal structure of the MMP-9, complexed with a reverse hydroxamate inhibitor (NFH), was retrieved from the Protein Data Bank (PDB ID: 1GKC) [27]. According to the docking results, NFH showed hydrogen bonds with Gly186, Leu188, Tyr421 (water mediated), Tyr423 and a π–sigma interaction with His401; these are in accordance with the X-ray structure binding features [27]. The reference drug NNGH showed hydrogen bonds with Leu188, Ala189, Glu402 and a π–π interaction with His401. The most potent MMP-9 inhibitor, compound **23**, which has carboxylate group as an ZBG, revealed hydrogen bonds with Gly186, Tyr423 and His401(Figure 3c). The van der Waals contact distances between the oxygen atoms of NFH and Zn atom are 2.04 and 1.956 Å (Figure 3b) while the van der Waals contact distances between the oxygen atoms of NNGH and Zn atom are 2.38 and 365 2.92 Å. Similarly, the van der Waals contact distance between the carboxylate oxygen of compound **23** and Zn atom is 1.978 Å (Figure 3c). Among all tested compounds **23** has the only carboxylate group on R position. According to docking results carboxylate group of **23** has a monodentate interaction with Zn atom, so this could explain it’s higher activity. When we look at the moderately active compound **22**, it showed hydrogen bonds with Gly186, Ala189 and the van der Waals contact distance between the carbonyl oxygen atom of amide function of **22** and Zn atom is 1.715 Å (Figure 3d). Compounds **17** and **18** have interactions with Zn atom similar to **22**. But both of them have only one hydrogen bond (water mediated) with the enzyme. Although they have interactions with Zn atom their activities are lower, because of the weak interaction with the enzyme MMP-9. Docking results of the other lower active compounds (**13**–**16**, **19**) showed H bonds with some of the active site residues but none of them has interaction with Zn atom.All of the docking results are given in Table 3.

## 3. Materials and Methods

### 3.1. General Information

All chemicals were from Sigma Aldrich Co. (St. Louis, MO, USA or Milan, Italy). Unless otherwise noted, reagents were obtained from commercial suppliers and were used without purification. Anhydrous toluene was obtained by distillation from Na, and anhydrous CH_2_Cl_2_ was obtained by distillation from calcium hydride. Melting points were measured on a Büchi 512 apparatus (Büchi Italia, Milano, Italy) and are uncorrected. The progress of the reactions was monitored by TLC with F254 silica-gel precoated sheets (Merck, Darmstadt, Germany). UV light was used for detection. Flash chromatography was performed using Merck silica gel 60 (Si 60, 40–63 µm, 230–400 mesh ASTM). Elemental analyses for C, H, and N were performed using a ThermoQuest Flash 1112 elemental analyzer (Termoquest Italia, Milano, Italy). The percentages found were within ±0.4% of the theoretical values. IR spectra were recorded on a Jasco FT-IR 460 plus spectrometer (Jasco Europe, Carpi, MO, Italy). ^1^H- and ^13^C-NMR spectra were recorded with an Avance 400 spectrometer (Bruker Italia, Milano, Italy) operating at 400 MHz and 100 MHz, respectively. Spectra were acquired from samples in DMSO-*d*_6_. Chemical shifts are reported as δ (ppm) relative to tetramethylsilane; coupling constants (*J*) are expressed in Hz. Mass spectra were recorded on a Thermo TSQ Quantum Access Max triple quadrupole mass spectrometer (Thermo, Waltham, MA, USA) equipped with a heated electrospray (H-ESI).

### 3.2. Chemistry

#### 3.2.1. General Procedure for the Synthesis of 2-(1,2-Benzothiazol-3-yl)-*N*′-(phenylmethylidene)-propanehydrazides **4**–**12**

The solution (or suspension for **11** and **12**) of 2-(1,2-benzothiazol-3-yl)propanoic acid hydrazide (1.35 mmol) and the suitable aldehyde (1.38 mmol) in 15 mL anhydrous ethanol, was refluxed for 8 h in the presence of acetic acid (0.25 mL). The mixture was cooled to room temperature, and the precipitate formed was filtered off, washed with water and then recrystallized from ethanol-water.

*2-(1,2-Benzothiazol-3-yl)-N′-(phenylmethylidene)propanehydrazide* (**4**), m.p.: 179–180 °C, yield 94%. FTIR (ΚBr, υ, cm^−1^): 3183 (NH), 3003–2932 (CH_3_), 1670 (C=O), 1648 (CH=N). ^1^H-NMR (DMSO-*d*_6_, δ, ppm): 11.45 (s, 1H, NH); 8.27–8.22 (m, 2H, Ar-4CH and Ar-7CH); 7.84 (s, 1H, CH); 7.65–7.56 (m, 2H, Ar-5CH and Ar-6CH); 7.22–7.18 (m, 4H, Ar-2’CH, Ar-6’CH, Ar-3’CH and Ar-5’CH); 5.22 (q, 1H, *J* = 7.2 Hz, CH); 1.54 (d, 1H, *J* = 7.2 Hz, CH_3_). Elemental Analysis for C_17_H_15_N_3_OS: Calcd. C, 66.00; H, 4.89; N, 13.58. Found: C, 65.05; H, 4.98; N, 13.39.

*2-(1,2-Benzothiazol-3-yl)-N′-[(4-methylphenyl)methylidene]propanehydrazide* (**5**), m.p.: 173–174 °C, yield 96%. FTIR (KBr, υ, cm^−1^): 3201 (NH), 3049–2983 (CH_3_), 1659 (C=O), 1547 (CH=N). ^1^H-NMR (DMSO-*d*_6_, *δ*, ppm):11.43 (s, 1H, NH); 8.27–8.16 (m, 2H, Ar-4CH and Ar-7CH); 7.80 (s, 1H, CH); 7.64–7.56 (m, 2H, Ar-5CH+Ar-6CH); 7.08 (d, 2H, *J* = 7.2 Hz, Ar-2’CH and Ar-6’CH); 7.00 (d, 2H, *J* = 7.6 Hz, Ar-3’CH and Ar-5’CH); 5.19 (q, 1H, *J* = 7.2 Hz, CH); 2.24 (s, 3H, CH_3_); 1.52 (d, 3H, *J* = 6.8 Hz, CH_3_). Elemental Analysis for C_18_H_17_N_3_OS: Calcd. C, 66.85; H, 5.30; N, 12.99. Found: C, 66.78; H, 5.28; N, 12.90.

*2-(1,2-Benzothiazol-3-yl)-N′-[(4-methoxyphenyl)methylidene]propanehydrazide* (**6**), m.p.: 198–199 °C, yield 83%. FTIR (KBr, υ, cm^−1^): 3201 (NH), 3059–2858 (CH_3_), 1663 (C=O), 1605 (CH=N). ^1^H-NMR (DMSO-*d*_6_, δ, ppm): 11.32 (s, 1H, NH); 8.26–8.16 (m, 2H, Ar-4CH and Ar-7CH); 7.80 (s, 1H, CH); 7.65–7.51 (m, 2H, Ar-5CH and Ar-6CH); 7.18 (d, 2H, *J* = 8.4 Hz, Ar-3’CH and Ar-5’CH); 6.77 (d, 2H, *J* = 8.8 Hz, Ar-2’CH and Ar-6’CH); 5.19 (q, 1H, *J* = 7.2 Hz, CH); 3.74 (s, 3H, CH_3_O); 1,53 (d, 3H, *J* = 6.8 Hz, CH_3_). Elemental Analysis for C_18_H_17_N_3_O_3_S_2_·0.25H_2_O (343.92): Calcd. C, 62.86.; H, 5.13; N, 12.22; Found C, 62.61; H, 4.92; N, 12.19%.

*2-(1,2-Benzothiazol-3-yl)-N′-[(4-fluorophenyl)methylidene]propanehydrazide* (**7**), m.p.: 184–185 °C, yield 89%. FTIR (KBr, υ, cm^−1^): 3185 (NH), 3043–2980 (CH_3_), 1661 (C=O), 1602 (CH=N). ^1^H-NMR (DMSO-*d*_6_, *δ*, ppm): 11.48 (s, 1H, NH); 8.26–8.16 (m, 2H, Ar-4CH and Ar-7CH); 7.84 (s, 1H, CH); 7.66–7.51 (m, 2H, Ar-5CH and Ar-7CH); 7.30–7.26 (m, 2H, Ar-2’CH and Ar-6’CH); 7.06–7.02 (m, 2H, Ar-3’CH and Ar-5’CH); 5.20 (q, 1H, *J* = 6.8 CH); 1.54 (d, 3H, *J* = 6.8 CH_3_). Elemental Analysis for C_19_H_17_N_3_O_3_S_2_·H_2_O (417.50): Calcd. C, 54.66.; H, 4.59; N, 10.06; Found C, 54.88; H, 4.06; N, 9.87%.

*2-(1,2-Benzothiazol-3-yl)-N′-{[4-(trifluoromethyl)phenyl]methylidene}propanehydrazide* (**8**), m.p.: 173–175 °C, yield 73%. FTIR (KBr, υ, cm^−1^): 3208 (NH), 2986–2890 (CH_3_), 1705 (C=O), 1605 (CH=N). ^1^H-NMR (DMSO-*d*_6_, *δ*, ppm): 11.74 (s, 1H, NH); 8.29–8.16 (m, 2H, Ar-4CH and Ar-7CH); 7.90 (s,1H, CH); 7.67–7.52 (m, 2H, Ar-5CH and Ar-7CH); 7.52 (d, 2H, *J* = 8.0 Hz, Ar-3’CH and Ar-5’CH); 7.40 (d, 2H, *J* = 8.4 Hz, Ar-2’CH and Ar-6’CH); 5,22 (q, 1H, *J* = 7.2 Hz, CH); 1.54 (d, 3H, *J* = 7.3 Hz, CH_3_). Elemental Analysis for C_18_H_14_F_3_N_3_OS (377.38): Calcd. C, 57.29.; H, 3.74; N, 11.13; Found C, 57.70; H, 3.83; N, 11.19%.

*2-(1,2-Benzothiazol-3-yl)-N′-[(4-chlorophenyl)methylidene]propanehydrazide* (**9**), m.p.: 238–240 °C, yield 77%. FTIR (KBr, υ, cm^−1^): 3148 (NH), 2982–2933 (CH_3_), 1698 (C=O), 1613 (CH=N). ^1^H-NMR (DMSO-*d*_6_, *δ*, ppm): 11.51 (S, 1H, NH); 8.22–8.16 (m, 2H, Ar-4CH and Ar-7CH); 7.82 (s, 1H, CH); 7.60–7.49 (m, 2H, Ar-6CH and Ar-5CH); 7.26–7.20 (m, 4H, Ar-2’CH, Ar-3’CH, Ar-5’CH and Ar-6’CH); 5.20 (q, 1H, *J* = 7.2 Hz, CH); 1.53 (d, 3H, *J* = 7.2 Hz, CH_3_). Elemental Analysis for C_17_H_14_ClN_3_OS·0.25H_2_O (348.34): Calcd. C, 58.62.; H, 4.19; N, 12.06; Found C, 58.54; H, 3.92; N, 12.07%.

*2-(1,2-Benzothiazol-3-yl)-N′-[(4-nitrophenyl)methylidene]propanehydrazide* (**10**), m.p.: 178–179 °C, yield 72%. FTIR (KBr, υ, cm^−1^): 3145 (NH), 2974–2932 (CH_3_), 1698 (C=O), 1513 (CH=N). ^1^H-NMR (DMSO-*d*_6_, *δ*, ppm): 11.76 (s, 1H, NH); 8.30–8.27 (m, 2H, Ar-4CH and Ar-7CH); 8.01 (d, 2H, *J* = 8.8 Hz, Ar-3’CH and Ar-5’CH); 7.93 (s, 1H, CH); 7.68–7.59 (m, 2H, Ar-5CH and Ar-6CH); 7.42 (d, 2H, *J* = 8.8 Hz, Ar-2’CH and Ar-6’CH); 5,24 (q, 1H, *J* = 7.0 Hz, CH); 1.55 (d, 3H, *J* = 7.2 Hz, CH_3_). Elemental Analysis for C_17_H_14_N_3_OS (348.34): Calcd. C, 57.62.; H, 3.98; N, 15.81; Found C, 57.35; H, 3.72; N, 15.53%.

*4-[{2-[2-(1,2-Benzothiazol-3-yl)propanoyl]hydrazinylidene}methyl]phenyl acetate* (**11**), m.p.: 168–170 °C, yield 88%. FTIR (KBr, υ, cm^−1^): 3184 (NH), 3010–2980 (CH_3_), 1766 (C=O), 1670 (C=O), 1647 (CH=N). ^1^H-NMR (DMSO-*d*_6_, *δ*, ppm): 11.47 (s, 1H, NH); 8.28–8.16 (m, 2H, Ar-4CH and Ar-7CH); 7.84 (s, 1H, CH); 7.66–7.50 (m, 2H, Ar-5CH and Ar-6CH); 7.23 (d, 2H, *J* = 8.4 Hz, Ar-3’CH and Ar-5’CH); 6.95 (d, 2H, *J* = 8.4 Hz, Ar-2’CH and Ar-6’CH); 5.20 (q, 1H, *J* = 7.0 Hz, CH); 2.25 (s, 3H, CO-CH_3_); 1.53 (d, 3H, *J* = 7.2 Hz, CH_3_). Elemental Analysis for C_19_H_17_N_3_OS (367.42): Calcd. C, 62.11.; H, 4.66; N, 11.44; Found C, 61.80; H, 4.73; N, 11.34%.

*Methyl 4-[{2-[2-(1,2-Benzothiazol-3-yl)propanoyl]hydrazinylidene}methyl]benzoate* (**12**), m.p.: 197–199 °C, yield 88%. FTIR (KBr, υ, cm^−1^): 3246 (NH), 2987–2945 (CH_3_), 1697 (C=O), 1594 (CH=N). ^1^H-NMR (DMSO-*d*_6_, *δ*, ppm): 11.63 (s, 1H, NH); 8.24–8.17 (m, 2H, Ar-4CH and Ar-7CH); 7.88 (s, 1H, CH); 7.72 (d, 2H, *J* = 8.4 Hz, Ar-3’CH and Ar-5’CH); 7.68–7,58 (m, 2H, Ar-5CH and Ar-6CH); 7,28(d, 2H, *J* = 8,4 Hz Ar-2CH and Ar-4CH); 5.21 (q, 1H, *J* = 6.8 Hz, CH); 3.83 (s, 3H, CH_3_); 1.53 (d, 3H, *J* = 7.2 Hz, CH_3_). Elemental Analysis for C_19_H_17_N_3_OS (367.42): Calcd. C, 62.11.; H, 4.66; N, 11.44; Found C, 61.75; H, 4.61; N, 11.52%.

#### 3.2.2. General Procedure for the Synthesis of 2-(1,2-Benzothiazol-3-yl)-*N*-(4-oxo-2-phenyl-1,3-thiazolidin-3-yl)Propanamides **13**–**19**

A mixture of appropriate hydrazides **4**–**10** (1.1 mmol) and sulfanylacetic acid (2.2 mmol) was refluxed for 48 h in 20 mL dry toluene under a nitrogen atmosphere. The reaction mixture was then cooled to room temperature and the solid precipitated was filtered off and washed with toluene (or filtered, after the mixture was concentrated in vacuo (**19**), and then taken up with an aqueous solution of 10% Na_2_CO_3_ and washed with water, as for **18**). The crude product was then purified by flash chromatography and recrystallized or directly recrystallized.

*2-(1,2-Benzothiazol-3-yl)-N-(4-oxo-2-phenyl-1,3-thiazolidin-3-yl)propanamide* (**13**), m.p.: 239–240 °C (EtOH-H_2_O), yield 44%. FTIR (KBr, υ, cm^−1^): 3221 (NH), 3036–2938 (CH_3_), 1714 (C=O), 1663 (CH=N). ^1^H-NMR (DMSO-*d*_6_, *δ*, ppm): 10.68 (s, 1H, NH); 8.15 (d, 1H, *J* = 8.0 Hz, Ar-4CH); 7.69 (d, 1H, *J* = 8.4 Hz, Ar-7CH); 7.57 (t, 1H, *J* = 7.8 Hz, Ar-5CH); 7.39–7.32 (m, 5H, Ar-2’CH, Ar-3’CH, Ar-4’CH, Ar-5’CH and Ar-6’CH); 7.25 (t, 1H, *J* = 7.4 Hz, Ar-6CH); 5.17 (s, 1H, CH); 4.27 (q, 1H, *J* = 6.9 Hz, CH); 3.89 (d, 1H, *J* = 16.0 Hz, CH-H); 3.75 (d, 1H, *J* = 15.6 Hz, CH-H); 1.55 (d, 3H, *J* = 6.8 Hz, CH_3_); ^13^C-NMR (DMSO-*d*_6_, *δ*, ppm): 170.84 (C=O); 169.38 (C=O); 163.51 (C=N); 152.27 (C); 138.49 (C); 133.89 (C); 129.53 (CH); 129.04 (CH); 128.24 (CH); 125.24 (CH); 122.35 (CH); 121.01 (CH); 61.99 (CH-); 41,11 (CH-); 29.77 (CH_2_); 16.05 (CH_3_); Negative EIMS *m*/*z* 382 [M]^−1^. Elemental analysis for C_19_H_17_N_3_O_2_S_2_: Calcd. (383.49): C, 59.51; H, 4.47; N, 10.96; Found C, 59.71; H, 4.41; N, 10.81%.

*2-(1,2-Benzothiazol-3-yl)-N-[2-(4-methylphenyl)-4-oxo-1,3-thiazolidin-3-yl]propanamide* (**14**), Flash chromatography; CH_2_Cl_2_/MeOH(NH_3_), 98:2. m.p.: 218–220 °C (toluene-EtOH), yield 65%. FTIR (KBr, υ, cm^−1^): 3244 (NH), 3031–2983 (CH_3_), 1713 (C=O), 1669 (CH=N). ^1^H-NMR (DMSO-*d*_6_, *δ*, ppm): 10.64 (s, 1H, NH); 8.16 (d, 1H, *J* = 8.4 Hz, Ar-4CH); 7.70 (d, 1H, *J* = 8.0 Hz, Ar-7CH); 7.58 (t, 1H, *J* = 7.2 Hz, Ar-5CH); 7.33–7.14 (m, 5H, Ar-6CH,Ar-2’CH, Ar-6’CH, Ar-3’CH and Ar-5’CH); 5.71 (s, 1H, CH); 4.26 (q, 1H, *J* = 6.8 Hz, CH); 3.86 (d, 1H, *J* = 16.0 Hz, CH-H); 3.74 (d, 1H, *J* = 15.6 Hz, CH-H); 2.33 (s, 3H, CH_3_); 1.54 (d, 3H, *J* = 7.2 Hz, CH_3_); ^13^C-NMR (DMSO-*d*_6_, *δ*, ppm): 170.74 (C=O); 169.31 (C=O); 163.52 (C=N); 152.26( C); 138.99 (C); 135.32 (C); 133.88 (C); 129.55 (CH); 128.23 (CH); 125.20 (CH); 123.58 (CH); 120.99 (CH); 61.84 (CH-); 41,09 (CH-); 29.78 (CH_2_); 21.30 (CH_3_); 16.06 (CH_3_); Negative EIMS *m*/*z* 427 [M]^−1^. Elemental Analysis for C_19_H_16_N_4_O_4_S_2_ (428.49): Calcd. C, 5.25; H, 3.83; N, 13.07; Found C, 53.25; H, 3.83; N, 12.92%.

*2-(1,2-Benzothiazol-3-yl)-N-[2-(4-methoxyphenyl)-4-oxo-1,3-thiazolidin-3-yl]propanamide* (**15**); Flash chromatography; CH_2_Cl_2_/EtOH, 98:2. m.p.: 170 °C (EtOH-H_2_O), yield 43%. FTIR (KBr, υ, cm^−1^): 3264 (NH), 3017–2933 (CH_3_), 1721 (C=O), 1669 (CH=N). ^1^H-NMR (DMSO-*d*_6_, *δ*, ppm): 10.61 (s, 1H, NH); 8.11 (d, 1H, *J* = 8.0 Hz, Ar-4CH); 7.72 (d, 1H, *J* = 8.0 Hz, Ar-7CH); 7.58 (t, 1H, *J* = 7.6 HZ, Ar-5CH); 7.30 (t, 1H, *J* = 7.4 Hz Ar-6CH); 7.22 (d, 2H, *J* = 8.4 Hz, Ar-2’CH andAr-6’CH); 6.88 (d, 2H, *J* = 8.4 Hz, Ar-3’CH and Ar-5’CH); 5.66 (s, 1H, CH); 4.27 (q, 1H, *J* = 6.8 HZ, CH); 3.5 (d, 1H, *J* = 16.0 Hz, CH-H); 3.79 (s, 3H, CH_3_-O); 3.73 (d, 1H, *J* = 16.0 Hz, CH-H); 1.54 (d, 3H, *J* = 6.4 Hz, CH_3_); ^13^C-NMR (DMSO-*d*_6_, *δ*, ppm): 170.72 (C=O); 169.20 (C=O); 163.53 (C=N); 160.26 (C); 152.27( C); 133.90 (C); 129.88 (C); 129.80 (C); 128.24 (CH); 125.22(CH); 123.60 (CH); 120.99 (CH); 114.32 (CH); 61.72 (CH-); 55.72 (CH_3_O-); 41.10 (CH-); 29.82 (CH_2_); 21.30 (CH_3_); 16.06 (CH_3_); Negative EIMS *m*/*z* 412 [M]^−1^. Elemental Analysis for C_20_H_19_N_3_O_3_S_2_ (413.51): Calcd. C, 58.09; H, 4.63; N, 10.16; Found C, 57.98; H, 4.49; N, 10.22%.

*2-(1,2-Benzothiazol-3-yl)-N-[2-(4-fluorophenyl)-4-oxo-1,3-thiazolidin-3-yl]propanamide* (**16**), m.p.: 185–186 °C (EtOH-H_2_O), yield 87%. FTIR (KBr, υ, cm^−1^): 3230 (NH), 3031–2930 (CH_3_), 1721 (C=O), 1665 (CH=N). ^1^H-NMR (DMSO-*d*_6_, *δ*, ppm): 10.62 (s, 1H, NH); 8.16 (d, 1H, *J* = 8.0 Hz, Ar-2’CH and Ar-6’CH); 8.09 (d, 1H, *J* = 8.0 Hz, Ar-4CH); 7.74 (d, 1H, *J* = 8.0 Hz, Ar-7CH); 7.41 (t, 1H, *J* = 7.8 Hz Ar-5CH); 7.31 (t, 1H, *J* = 7.8 Hz, Ar-6CH); 7.24–7.14 (m, 2H, A-r3’CH and Ar-5’CH); 5.73 (s, 1H, CH); 4.25 (q, 1H, *J* = 7.2 Hz, CH); 3.89 (d, 1H, *J* = 1.,0 Hz, CH-H); 3.75 (d, 1H, *J* = 16.0 Hz, CH-H); 1.54 (d, 3H, *J* = 6.8 Hz, CH_3_); ^13^C-NMR (DMSO-*d*_6_, *δ*, ppm): 170.78 (C=O); 169.10 (C=O); 163.43 (C=N); 152.50 (C); 134.64 (C); 133.86 (C); 130.64 (C); 128.24 (CH); 125.17(C); 124.19 (CH); 123.55 (CH); 120.92 (CH); 115.82 (CH); 61.19 (CH-); 44.27 (CH-); 29.69 (CH_2_); 16.25 (CH_3_); Negative EIMS *m*/*z* 400 [M]^−1^. Elemental Analysis for C_19_H_16_FN_3_O_4_S_2_: Calcd. (401.48) C, 56.84.; H, 4.02; N, 10.47; Found C, 56.94; H, 3.92; N, 10.44%.

*2-(1,2-Benzothiazol-3-yl)-N-{4-oxo-2-[4-(trifluoromethyl)phenyl]-1,3-thiazolidin-3-yl}propanamide* (**17**), Flash chromatography; CH_2_Cl_2_/MeOH(NH_3_), 99:1. m.p.: 145–147 °C (EtOH-H_2_O), yield 25%. FTIR (KBr, υ, cm^−1^): 3220 (NH), 3028–2923 (CH_3_), 1714 (C=O), 1663 (CH=N). ^1^H-NMR (DMSO-*d*_6_, *δ*, ppm): 10.70 (s, 1H, NH); 8.15 (d, 1H, *J* = 8.4 Hz, Ar-4CH); 7.76–7.69 (m, 3H, Ar-7CH, Ar-3’CH andAr-5’CH); 7.57–7.53 (m,3H, Ar-5CH, Ar-2’CH and Ar-6’CH); 7.20 (td, 1H, *J* = 7.2, 1.2 Hz Ar-6CH); 5.82 (s, 1H, CH); 4.29 (q, 1H, *J* = 6.9 Hz, CH); 3.95 (dd, 1H, *J* = 16.0, 1.6 Hz CH-H); 3.78 (d, 1H, *J* = 15.6 Hz, CH-H); 1.54 (d, 3H, *J* = 7.2 Hz CH_3_); ^13^C-NMR (DMSO-*d*_6_, *δ*, ppm): 170.68 (C=O); 169.30 (C=O); 163.49 (C=N); 152.29 (C); 143.51 (C); 133.80 (C); 129.03 (CH); 128.22 (CH); 125.92(CH); 125.04 (CH); 123.48 (CH); 121.02 (CH); 61.03 (CH-); 41.09 (CH-); 29.64 (CH_2_); 15.98 (CH_3_); Negative EIMS *m*/*z* 450 [M]^−1^. Elemental Analysis for C_19_H_16_F_3_N_3_O_4_S_2_: Calcd. (451.49): C, 53.20.; H, 3.57 N, 9.31; Found C, 53.12; H, 3.38; N, 9.00%.

*2-(1,2-Benzothiazol-3-yl)-N-[2-(4-chlorophenyl)-4-oxo-1,3-thiazolidin-3-yl]propanamide* (**18**), m.p.: 187–188 °C (EtOH-H_2_O), yield 97%. FTIR (KBr, υ, cm-1): 3219 (NH), 3029–2935 (CH3), 1718 (C=O), 1664 (CH=N). ^1^H-NMR (DMSO-*d*_6_, *δ*, ppm): 10.65 (s, 1H, NH); 8,16 (d, 1H, *J* = 8.0 Hz, Ar-4CH); 7.74 (d, 1H, *J* = 8.0 Hz, Ar-7CH); 7.58 (t, 1H, *J* = 7.6 Hz, Ar-5CH); 7.39 (d, 2H, *J* = 8.8 Hz, Ar-3’CH and Ar5’CH); 7.32 (d, 2H, *J* = 8.4 Hz, Ar-2’CH and Ar-6’CH); 7.28 (t, 1H, *J* = 7.6 Hz, Ar-6CH); 5.73 (s, 1H, CH); 4.28 (q, 1H, *J* = 7.2 Hz, CH); 3.90 (d, 1H, *J* = 16.0 Hz, CH-H); 3.76 (d, 1H, *J* = 16.0 Hz, CH-H); 1.54 (d, 3H, *J* = 6.8 Hz, CH_3_); ^13^C-NMR (DMSO-*d*_6_, *δ*, ppm): 170.65 (C=O); 169.19 (C=O); 163.49 (C=N); 152.28 (C); 137.58; (C); 134.04 (C); 133.84 (C); 130.17 (CH); 128.98 (CH); 128.23(CH); 125.17 (CH); 123.53 (CH); 121.01 (CH); 61.14 (CH-); 41.10 (CH-); 29.69 (CH_2_); 16.03 (CH_3_); Negative EIMS *m*/*z* 416 [M]^−1^. Elemental Analysis for C_19_H_16_ClN_3_O_2_S_2_ (417.93): Calcd. C, 54.60; H, 3.86; N, 10.05; Found C, 54.26; H, 3.65; N, 9.97%.

*2-(1,2-Benzothiazol-3-yl)-N-[2-(4-nitrophenyl)-4-oxo-1,3-thiazolidin-3-yl]propanamide* (**19**), Flash chromatography; CH_2_Cl_2_/EtOH (98:2). m.p.: 208–209 °C (EtOH-H_2_O), yield 88%. FTIR (KBr, υ, cm^−1^): 3247 (NH), 3030–2920 (CH_3_), 1719 (C=O), 1664 (CH=N).^1^H-NMR (DMSO-*d*_6_, *δ*, ppm): 10.72 (s, 1H, NH); 8.14–8.12 (m, 3H, Ar-4CH, Ar-5CH, Ar-3’CH and Ar-5’CH); 7.76 (d, 1H, *J* = 8.4 Hz, Ar-7CH); 7.56 (d, 2H, *J* = 8.4 Hz, Ar-2’CH and Ar-6’CH); 7.52 (t, 1H, *J* = 7.6 Hz, Ar-5CH); 7.22 (t, 1H, *J* = 7.6 Hz, Ar-6CH); 5.88 (s, 1H, CH); 4.29 (q, 1H, *J* = 7.0 Hz, CH); 3.95 (d, 1H, *J* = 16.0 Hz, CH-H); 3.80 (d, 1H, *J* = 16.0 Hz, CH-H); 1.51 (d, 3H, *J* = 7.0 Hz, CH_3_); ^13^C-NMR (DMSO-*d*_6_, *δ*, ppm): 170.52 (C=O); 169.20 (C=O); 163.47 (C=N); 152.25 (C); 148.08 (C); 146.23 (C);133.76 (C); 129.45 (CH); 128.16(CH); 125.10 (CH); 124.05 (CH); 123.53 (CH); 120.97 (CH); 60.61 (CH-); 41,07 (CH-); 29.61 (CH_2_); 15.96 (CH_3_); Negative EIMS *m*/*z* 427 [M]^−1^. Elemental Analysis for C_19_H_16_N_4_O_4_S_2_ (428.49): Calcd. C, 53.25; H, 3.83; N, 13.07; Found C, 53.25; H, 3.83; N, 12.92%.

#### 3.2.3. Synthesis of 3-{[2-(1,2-Benzothiazol-3-yl)propanoyl]amino}-1,3-thiazolidin-4-ones **20**–**21**

A mixture of the hydrazide **11** or **12** (1.1mmol) and sulfanylacetic acid (2.2 mmol) was refluxed for 48 h in dry toluene (20 mL), under a nitrogen atmosphere. After cooling toroom temperature, the solvent was evaporated, and the oily residue was taken up with an aqueous solution of 10% Na_2_CO_3_ and extracted (3×) with EtOAc. The organic phases were combined, dried (Na_2_SO_4_), and evaporated to afford a crude product which was purified by flash chromatography over silica gel (CH_2_Cl_2_/MeOH(NH_3_), 99:1 as eluent) and recrystallized. 

*4-(3-{[2-(1,2-Benzothiazol-3-yl)propanoyl]amino}-4-oxo-1,3-thiazolidin-2-yl)phenyl acetate* (**20**), m.p.: 120 °C (EtOH-H_2_O), yield 14%. FTIR (KBr, υ, cm^−1^): 3224 (NH), 3003–2935 (CH_3_), 1747 (C=O), 1663 (CH=N). ^1^H-NMR (DMSO-*d*_6_, *δ*, ppm): 10.73 (s, 1H, NH); 8.15 (d, 1H, *J* = 8.4 Ar-4CH); 7.69 (d, 1H, *J* = 8.4 Hz, Ar-7CH); 7.56 (t, 1H, *J* = 7.2, Ar-5CH); 7.37 (d, 2H, *J* = 8.8, Ar-2’CH and Ar-5’CH); 7.29 (t, 1H, *J* = 7.2, Ar-6CH); 7.14 (d, 2H, *J* = 8.8, Ar-2’CH and Ar-6’CH); 5.73 (s, 1H, CH); 4.28 (q, 1H, *J* = 7.2, CH); 3.90 (dd, 1H, *J* = 16.0, 1.6, Hz, CH-H); 3.75 (d, 1H, *J* = 15.6 Hz, CH-H); 2.31 (s, 3H, CH_3_); 1.55 (d, 3H, *J* = 7.2 Hz, CH_3_). ^13^C-NMR (DMSO-*d*_6_, *δ*, ppm): 170.88 (C=O); 169.84 (C=O); 169.82 (C=O); 165.45 (C=N); 152.84 (C); 152.65 (C) 134.64; (C); 133.86 (C); 130.64 (C); 128.24 (CH); 125.17(C); 124.19 (CH); 123.55 (CH); 120.92 (CH); 115.82 (CH); 61.19 (CH-); 44,27 (CH-); 29.69 (CH_2_); 16.25 (CH_3_); Negative EIMS *m*/*z* 440 [M]^−1^. Elemental Analysis for C_21_H_19_N_3_O_4_S_2_ (441.52): Calcd. C, 57.36.; H, 4.34; N, 9.52; Found C, 57.36; H, 4.08; N, 9.26%.

*Methyl 4-(3-{[2-(1,2-benzothiazol-3-yl)propanoyl]amino}-4-oxo-1,3-thiazolidin-2-yl)benzoate* (**21**), m.p.: 144–145 °C (EtOH-H_2_O), yield 34%. FTIR (KBr, υ, cm^−1^): 3264 (NH), 3035–2917 (CH_3_), 1708 (C=O), 1664 (CH=N). ^1^H-NMR (DMSO-*d*_6_, *δ*, ppm): 10.71 (s, 1H, NH); 8.14 (d, 1H, *J* = 8.4 Hz, Ar-4CH); 7.88 (d, 2H, *J* = 8.4 Hz, Ar-3’CH and Ar-5’CH); 7.69 (d, 1H, *J* = 8.1 Hz, Ar-7CH); 7.54 (td, 1H, *J* = 7.7, 0.9 Hz, Ar-5CH); 7.43 (d, 2H, *J* = 8.4 Hz, Ar-2’CH and Ar-6’CH); 7.19 (td, 1H, *J* = 7.4, 0.9 Hz, Ar-6CH); 5.80 (s, 1H, CH); 4.27 (q, 1H, *J* = 7.2 Hz, CH); 3.96–3.88 (m, 4H, CH-H and CH_3_O); 3.78 (d, 1H, *J* = 15.6 Hz, CH-H); 1.52 (d, 3H, *J* = 6.9 Hz, CH_3_). ^13^C-NMR (DMSO-*d*_6_, *δ*, ppm): 170.88 (C=O); 169.64 (C=O); 169.32 (C=O); 152.24 (C=N); 152.37 (C); 152.65 (C); 136.01 (C); 133.89 (C); 129.46 (C); 128.27 (CH); 123.51 (CH); 122.47 (CH); 121.01 (CH); 120.92 (CH); 61.41 (CH-); 40.18 (CH-); 29.74 (CH_3_O-); 21.37 (CH_2_); 16.25 (CH_3_); Negative EIMS *m*/*z* 440 [M]^−1^. Elemental Analysis for C_21_H_19_N_3_O_4_S_2_·0.25H_2_O (446.03): Calcd. C, 56.65; H, 4.41; N, 9.42; Found C, 56.34; H, 4.33; N, 9.42%.

#### 3.2.4. Synthesis of 2-(1,2-Benzothiazol-3-yl)-1,3-thiazolidin-4-one Target Derivatives **22**–**23**

To a stirred solution of the appropriate above 3*-{[2-(1,2-benzothiazol-3-yl)propanoyl]am*ino}-1,3-thiazolidin-4-one **20** or **21** (0.6 mmol) in ethanol (30 mL) a 15% aqueous solution of NaOH (18 mL) was addedand the resulting solution was stirred at room temperature for 2 h. The ethanol was evaporated under reduced pressure, and then 1N HCl was added (to acidic pH), providing a white solid that was filtered off, washed with water and then recrystallised from ethanol-water.

*2-(1,2-Benzothiazol-3-yl)-N-[2-(4-hydroxyphenyl)-4-oxo-1,3-thiazolidin-3-yl]propanamide* (**22**), m.p.: 257–259 °C, yield 62%. FTIR (KBr, υ, cm^−1^): 3255 (NH), 2985–2935 (CH_3_), 1721 (C=O), 1681 (CH=N). ^1^H-NMR (DMSO-*d*_6_, *δ*, ppm): 10.58 (s, 1H, NH); 9.66 (s, 1H, OH); 8.16 (d, 1H, *J* = 8.4 Hz, Ar-CH4); 7.72 (d, 1H, *J* = 8.0 Hz, Ar-7-CH); 7.59 (t, 1H, *J* = 7.2 Hz, A-5CH); 7.30 (t, 1H, *J* = 7.2 Hz Ar-6CH); 7.12 (d, 2H, *J* = 8.8 Hz, Ar-2’CH and Ar-6’CH); 6.73 (d, 2H, *J* = 8.4 Hz, Ar-3’CH and Ar-5’CH); 5.61 (s, 1H, CH); 4.26 (q, 1H, *J* = 6.8 Hz, CH); 3.82 (dd, 1H, *J* = 15.0, 1.2 Hz, CH-H); 3.71 (d, 1H, *J* = 15.0 Hz, CH-H); 1.54 (d, 3H, *J* = 6.4 Hz, CH_3_); ^13^C-NMR (DMSO-*d*_6_, *δ*, ppm): 170.78 (C=O); 169.18 (C=O); 163.54 (C=O); 158.65 (C); 152.24 (C=N); 133.92 (C); 129.86 (CH); 128.25 (CH); 127.98 (CH); 125.22 (CH); 123.61 (CH); 121.01 (CH); 115.72 (CH); 61.99 (CH-); 41.10 (CH-); 29.83 (CH_2_); 16.10 (CH_3_); Negative EIMS *m*/*z* 398 [M]^−1^. Elemental Analysis for C_19_H_17_N_3_O_3_S_2_·H_2_O (417.50): Calcd. C, 54.66.; H, 4.59; N, 10.06; Found C, 54.88; H, 4.06; N, 9.87%.

*4-(3-{[2-(1,2-Benzothiazol-3-yl)propanoyl]amino}-4-oxo-1,3-thiazolidin-2-yl)benzoic acid* (**23**), m.p.: 137–140 °C, yield 90%. TLC; FTIR (KBr, υ, cm^−1^): 3248 (NH), 3005–2941 (CH_3_), 1722 (C=O), 1667 (CH=N). ^1^H-NMR (DMSO-*d*_6_, *δ*, ppm): 13.08 (s, 1H, COOH); 10.70 (s, 1H, NH); 8.14 (d, 1H, *J* = 8.4 HZ, Ar4); 7.89 (d, 2H, *J* = 8.4 Hz, Ar3’+5’); 7.70 (d, 1H, *J* = 8.4 HZ, Ar7); 7.54 (t, 1H, *J* = 7.4 Hz, Ar5); 7.43 (d, 2H, *J* = 8.4 Hz, Ar2’+6’); 7.20 (t, 1H, *J* = 7.4 Hz, Ar6); 5.79 (s, 1H, CH); 4.28 (q, 1H, *J* = 7.2 Hz, CH); 4.28 (q, 1H, *J* = 7.2 Hz, CH); 3.92 (dd, 1H, *J* = 16.1, 1.3 Hz, CH-H); 3.77 (d, 1H, *J* = 16.0 Hz, CH-H); 1.54 (d, 3H, *J* = 6.8 Hz, CH_3_); ^13^C-NMR (DMSO-*d*_6_, *δ*, ppm): 170.71 (C=O); 169.31 (C=O); 167.37 (C=O); 163.49 (C); 152.27 (C=N); 143.44 (C); 133.82 (C); 131.77 (C); 129.98 (CH); 128.34 (CH); 128.20 (CH); 125.10 (CH); 123.48 (CH); 121.01 (CH); 61.31 (CH-); 41.01 (CH-); 29.68 (CH_2_); 16.03 (CH_3_); Negative EIMS *m*/*z* 426 [M]^−1^. Elemental Analysis for C_20_H_17_N_3_O_4_S_2_·0.5H_2_O (436.51): Calcd. C, 55.03; H, 4.16; N, 9.63; Found C, 55.03; H, 4.01; N, 9.68%.

### 3.3. Oxygen Radical Absorbance Capacity (ORAC) Assays

The ORAC assay was performed as previously reported by Cao et al. [28]. The measurements were carried out on a Wallac 1420 Victor^3^ 96-well plate reader (EG & Wallac, Turku, Finland) with fluorescence filter (excitation 540 nm/8 nm, emission 570 nm/7 nm). Fluorescein (10 nM) was the fluorescence probe and target molecule for free radical attack from AAPH (100 mM) as peroxyl radical generator. The reaction was conducted at 37 °C at pH 7.0 with Trolox (12.5 μM) as control standard and phosphate buffer as blank. Compounds **13**–**19** and **22**–**23** were appropriately diluted with DMSO/buffer (1:10) prior to analysis. The fluorescein fluorescence was recorded every 2 min after addition of AAPH. All measurements were expressed relative to the initial reading. One blank, one standard, and a maximum of 10 samples were analyzed at the same time. Each measure was repeated at least three times. The ORAC value refers to the net protection area under the quenching curve of fluorescein in the presence of an antioxidant. The final results (ORAC value) were calculated and expressed using Trolox equivalents (TE) per gram of extract (TE/g):

ORAC value (μM) = K(S sample − S blank)/(S Trolox − S blank)(1)

where K is a sample dilution factor, S is the area under the fluorescence decay curve of the sample, Trolox or blank. This area was calculated with Origin^®^7 (OriginLab Corporation, Northampton, MA, USA).

### 3.4. MMP-9 Fluorimetric Assay 

The compounds were evaluated for their ability to inhibit the hydrolysis of fluorescence-quenched peptide substrate Mca-Pro-Leu-Gly-Leu-Dpa-Ala-Arg-NH_2_ (Biomol, Inc., Enzo Life Sciences, Inc., Farmingdale, NY, USA). The MMP-9 assays were performed in 50 mM HEPES buffer containing 5 mM CaCl_2_, 0.1 mM ZnCl_2_, 0.05% Brij-35, at pH 7, using 10 nM of proteolytic enzyme (catalytic domains of MMP-9 (Biomol, Inc.) and 350 nM of peptide substrate. The enzyme was incubated at 25 °C with increasing concentration (0.0004, 0.004, 0.04, 0.4, 4, 40, 400 µM) of compounds, and the fluorescence (excitation max 328 nm; emission max 393 nm) was measured for 3 min after the addition of the substrate using a Victor Wallac 1420 Multilabel Counters fluorimeter (Perkin Elmer, Waltham, MA, USA). The fluorescence values of the samples were plotted against the time with a linear regression fit, using the Origin software, version 7 (Origin Lab Corporation, Northampton, MA, USA). The slope of the line value for each inhibitor concentrations was obtained where an increase of the slope corresponds to a decrease of inhibitory activity. Subsequently, the IC_50_ values were obtained from a non-linear regression procedure (log inhibitor vs. response) with a variables slope of the regression line (Y) and the molar inhibitor concentration (X), using the software Origin^®^7 (Origin Lab Corporation). The IC_50_ value was obtained from the equation Y = P1/(1 + X/P2). NNGH (*N*-Isobutyl-*N*-(4-methoxyphenylsulfonyl)glycylhydroxamic acid) a potent MMPs inhibitor was used as a positive control [29].

### 3.5. Keratinocyte Cultures and Treatments

The normal human keratinocyte cell line NCTC 2544 was provided by Interlab Cell Line Collection Genoa (Genova, Italy) and cultured in Minimum Essential Medium (MEM) (Sigma-Aldrich, Milan, Italy) containing 10% fetal calf serum, 100 U/mL penicillin and 100 μg/mL streptomycin at 37 °C in a humidified, 95% air/5% CO_2_ atmosphere. The medium was changed every 2–3 days. A day before the experiment, cells were trypsinized, counted, and plated either in 96 wells or in 6 well plates. Experimental keratinocytes were stimulated or not (untreated controls) with 200 U/mL of IFN-γ and 10^−4^ M of histamine (H) in presence or absence of different concentrations of EM compounds (10, 50 and 100 μM). After 48 h each sample was tested for the experiments described below.

### 3.6. Cell Viability Assay

Cell viability was evaluated by determining mitochondrial function of living cells on the basis of their ability to reduce the yellow dye, tetrazolium salt 3-(4,5-dimethylthiazol-2-yl)-2,5-diphenyltetrazolium bromide (MTT) (Sigma-Aldrich, Milan, Itlay), into dark blue formazan crystal mainly by the mitochondrial dehydrogenases [30]. Cells were seeded at the density of 8.5 × 10^3^ cells/well in 96-wells plates and incubated at 37 °C in an atmosphere of 5% CO_2_ for 24 h with growing medium. At this time, NCTC 2544 cells were stimulated with 200 U/mL of IFN-γ and 10^−4^ M of H and treated with different concentrations (10, 50 and 100 μM) of compounds. After 48 h, 200 μL of MTT in PBS at final concentration of 0.5 mg/mL were added into each well for 3 h at 37 °C. Next, the blue formazan crystals were solubilized with 100 μL of dimethylsulphoxide (DMSO); a microplate spectrophotometer reader (Titertek Multiskan, DAS, Palombara Sabina, Italy) at λ = 550 nm measured the absorbance. For each sample, three experiments in triplicate were performed and percentage of cell viability of treated cells was compared with that of untreated control cells.

### 3.7. Western Blot Analysis

The expression of nuclear factor kappa-light-chain-enhancer of activated B cells (NF-κB) was evaluated by Western blot analysis. Treated and untreated cells were trypsinized, pelletted and washed three times with PBS. Proteins were extracted by M-PER^®^ Mammalian Protein Extraction Reagent (Thermo Scientific, Pierce Biotechnology, Waltham, MA, USA) supplemented with a cocktail of protease inhibitors (complete, Mini, Protease Inhibitor Cocktail Tablets, Roche, Basel, Switzerland) according to manufacturer’s instructions and the protein concentration was estimated using the Bicinchoninic acid assay (Pierce). Equal amount of proteins was boiled in LDS sample buffer (Invitrogen, Carlsbad, CA, USA) in presence of 1X sample reducing agent (Invitrogen). Each sample was then subjected to electrophoresis on Bolt™ 4–12% Bis-Tris Plus Gels (Invitrogen). After electrophoresis, proteins were transferred to nitrocellulose membrane, in a wet system, and proteins transfer was verified by staining membranes with Ponceau S. Membranes were blocked with Tris buffered saline containing 0.01% Tween-20 (TBST) and 5% non-fat dry milk for 1 h RT, and then probed overnight at 4 °C with the following primary antibodies: anti-NF-κB p-50 and anti-β-actin.The membranes were rinsed three times in TBST and the appropriate HRP-conjugated secondary antibody was incubated for 1 h at RT. The blots were developed using enhanced chemiluminescent solution (Millipore, Billerica, MA, USA) and visualized with a chemiluminescent western blot imaging system (Alliance, UVITEC, Ltd., Cambridge, UK). Bands were measured densitometrically and their relative density was calculated based on the density of the ^®^-actin in each sample. Results were expressed as arbitrary densitometric units (A.D.U.) corresponding to signal intensity with respect to loading control.

### 3.8. Statistical Analysis

All the present results are means S.E.M. of three experiments performed on quadruplicate samples. The Student’s *t*-test was used to evaluate the differences between the means of each group. *P* < 0.05 was considered to be statistically significant. The statistical analysis was performed by using one-way ANOVA followed by Dunnett’s post-hoc test for multiple comparison with control. All statistical analyses were performed using the statistical software package SYSTAT, version 9 (Systat Inc., Evanston, IL, USA).

### 3.9. DockingStudies

#### 3.9.1. Preparation of the Enzyme

The crystal structure of the MMP-9, complexed with a reverse hydroxamate inhibitor (NFH) was retrieved from the Protein Data Bank (PDB ID: 1GKC) [27]. Accelrys Discovery Studio 3.5 [22] software (BIOVIA, San Diego, CA, USA) was used for preparation of protein and ligands. The target protein was taken, the ligand was extracted, hydrogens were added and their positions were optimized using the all atom CHARMm forcefield and the Adopted Basis set Newton Raphson (ABNR) method available in Discovery Studio 3.5 protocol until the root mean deviation (RMS) gradient was <0.05 kcal/mol/Å^2^. The minimized protein was defined as the receptor using the binding site module. The binding site was defined from the cavity finding method which was modified to accommodate all the important interacting residues in the active site. Binding sphere for 1GKC (65.86, 30.94, 118.05, 9.78) was selected from the active site using the binding site tools.

#### 3.9.2. Preparation of Ligands

Novel synthesized 4-thiazolidinone derivatives **13**–**19** and **22**–**23**, NFH and standard drug NNGH were sketched, all atom CHARMm forcefield parameterization was assigned and then minimized using the ABNR method as described above. Conformational searches of the ligands were carried out using a simulated annealing molecular dynamics (MD) approach. The ligands were heated to a temperature of 700 K and then annealed to 200 K.

#### 3.9.3. Molecular Docking

CDocker [31] method was performed by using Discovery Studio 3.5. The protein is held rigid while the ligands are allowed to be flexible during refinement. The docking parameters were as follows: Top Hits: 10; Random Conformations: 10; Random Conformations Dynamics Step: 1000; Grid Extension: 8.0; Random Dynamics Time Step: 0.002. The docking and scoring methodology was first validated by docking of NFH. The docked position of NFH overlaps well with the crystal structure position, with an RMSD of 0.29 Å. Afterwards molecular docking studies were performed on the new synthesized compounds.

#### 3.9.4. Analysis of Results

Finally, all docked poses were scored by applying Analyze Ligand Poses subprotocol and binding energies were calculated by applying Calculate Binding Energy subprotocol in Discovery Studio 3.5 by using in situ ligand minimization step (ABNR method) and using implicit solvent model (GBMV). The lowest binding energy was taken as the best-docked conformation of the compound for the macromolecule.

## 4. Conclusions

On the whole our results indicate that the appreciable anti-inflammatory/potential wound healing effects of the compounds tested are supported by the 4-thiazolidinone core, the benzisothiazole system and the isopropanoylhydrazide spacer, whereas the 4-phenyl substituent at C2 of the 4-thiazolidinone ring appears to be able to modulate the activity. Among the tested compounds derivative **23**, bearing a 4-carboxyphenyl substituent at C2 of the 4-thiazolidinone ring, exhibited the most promising profile, being able to inhibit MMP-9 at nanomolar level (IC_50_ = 40 nM) and to reduce NF-κB levels (50% at 10 µM). Accordingly, **23** shows good antioxidant activity (ORAC value = 0.53 TE/µmol). Docking studies, performed as reported above, show that carboxylate group of 23 has a monodentate interaction with Zn atom and H bonds with three of the active site residues (Gly186, Tyr423 and His401) that could explain its higher activity. 4-(3-{[2-(1,2-Benzothiazol-3-yl)propanoyl]amino}-4-oxo-1,3-thiazolidin-2-yl)benzoic acid (**23**) can therefore be considered as a lead compound for the development of new therapeutic agents to prevent tissue damage.

## Figures and Tables

**Table 1:** Antioxidant activity of 4-thiazolidinones **13**–**19**, **22** and **23**.

Compounds		ORAC UNITs ^a^
**13**	R=-H	0.61 ± 0.14 *
**14**	R=-CH_3_	0.40 ± 0.06 *
**15**	R=-OCH_3_	0.66 ± 0.12 *
**16**	R=-F	0.46 ± 0.20 *
**17**	R=-CF_3_	0.27 ± 0.06 *
**18**	R=-Cl	0.47 ± 0.23 *
**19**	R=-NO_2_	0.40 ± 0.17 *
**22**	R=-OH	1.24 ± 0.09 *
**23**	R=-COOH	0.53 ± 0.17 *

^a^ ORAC values are reported as ORAC unit, corresponding to μmol of Trolox equivalents (TE) per micromol (µmol) of sample. Trolox = 1 ORAC Units. Reported values are the means ± Standard deviation (SD) (*n* = 3). * Significantly different at *p* < 0.5 compared to Trolox.

**Table 2:** In vitro inhibitory activity of 4-thiazolidinones **13**–**19**, **22** and **23** toward MMP-9.

Compounds		IC_50_ Value (µM)
**13**	R=-H	7.99 ± 0.65 *
**14**	R=-CH_3_	9.01 ± 0.45 *
**15**	R=-OCH_3_	6.82 ± 0.95 *
**16**	R=-F	17.15 ± 1.92 *
**17**	R=-CF_3_	n.a.
**18**	R=-Cl	16.12 ± 1.23 *
**19**	R=-NO_2_	22.05 ± 1.42 *
**22**	R=-OH	0.30 ± 0.05 *
**23**	R=-COOH	0.04 ± 0.01 *
NNGH ^		0.0065 ± 0.00025

n.a. = not active; ^ NNGH ((*N*-Isobutyl-*N*-(4-methoxyphenylsulfonyl)glycylhydroxamic acid) is the reference standard of assay; Reported values are the means ± Standard Deviation (SD) (*n* = 3); * *p* < 0.05 compared to NNGH value.

**Table 3:** Interacted residues of the tested compounds according to the docking results.

Compound	Interacted Residues *	Binding Energies
**13**	Gly186, Leu187, **Leu188** (1.66 Å), Ala189, His190, Ala191, Tyr393, Val398, His401, **Glu402** (2.40 Å), His405, His411, Pro421, Met422, **Tyr423** (1.89 Å)	−22.10
**14**	Gly186, Leu187, **Leu188** ^[b]^ (1.92 Å), Ala189, Tyr393, Leu397, Val398, His401 ^[a]^, Glu402, Leu418, Tyr420, **Pro421** (2.30 Å), Met422, Tyr423, Arg424	−21.30
**15**	Gly186, Leu187, **Leu188** (2.19 Å), **Ala189** (2.13 Å), His190, Tyr393, Leu397, Val398, His401 ^[a]^, Glu402, Leu418, Tyr420, Pro421, Met422, **Tyr423** (2.28 Å), Arg424	−22.33
**16**	Gly186, Leu187, **Leu188** (1.73 Å), Ala189 (2.18 Å), His190, Ala191, Tyr393, Val398, His401, **Glu402** (2.44), His405, Pro421, Met422, **Tyr423** (1.48 Å)	−17.50
**17**	Phe110, Glu111, Leu187, Leu188, Ala189, His190, **Ala191** (2.49 Å) (water mediated), Val398, His401 ^[a]^, Glu402, His405, His411, Tyr420, Pro421, Met422, Tyr423, Zn	−24.19
**18**	Phe110, Glu111, Gly186, Leu187, Leu188, Ala189, His190, **Ala191** (2.37 Å) (water mediated) His401, Glu402, His405, His411, Pro421, Met422, Tyr423, Zn	−32.33
**19**	Gly186, Leu187, Leu188, **Ala189** (2.24 Å), His190, Val398, His401, Glu402, His405, His411, Pro421, Met422, Tyr423	−18.63
**22**	Phe110, Glu111, **Gly186** (1.75 Å), Leu187, Leu188, **Ala189** (2.12 Å), His190, Ala191, Val398, His401, Glu402, His405, His411, Pro421, Met422, Tyr423, Zn	−24.70
**23**	Asp185, **Gly186** (2.03 Å), Leu187, Leu188, Tyr393, **His401** (2.26 Å), Glu402, His411, Pro421, Met422, **Tyr423** (2.35 Å), Zn	−24.30
**NFH**	**Gly186** (2.32), Leu187, **Leu188** (1.92 Å), Ala189, His190, Ala191, Tyr393, Val398, His401 ^[b]^, Glu402, His405, His411, Tyr420, **Pro421** (2.04 Å) (water mediated), Met422, **Tyr423** (2.15 Å), Zn	−70.88
**NNGH**	Gly186, Leu187, **Leu188** (2.17 Å), **Ala189** (2.19 Å), His190, Ala191, Leu397, Val398, His401 ^[a]^, **Glu402** (1.34 Å), His405, His411, Leu418, Tyr420, Pro421, Met422, Tyr423, Arg424, Zn	−47.76

**Bold:** H-bonds; ^[a]^: π–π interactions; ^[b]^: π–sigma interactions; * van der Waals contact distance <4 Å.

## Supplementary Materials

The supplementary materials are available. The representative dose-response curves for IC_50_ value determination of tested compounds on MMP-9 are showed in Figures S1–S8. The logarithm value of each increasing μmolar (µM) concentration values of compounds are reported in the *X* axes. The logarithm value of 0.0004 mM concentration (−7) corresponds to a very low dose that gives the same response of blank. With the term “blank” on the left to indicate absence of the inhibitor. The increasing slope of the regression line value, which corresponds relating to a decrease in the inhibitory activity, was reported in the *Y* axes. The values between the points were determined conducting a non linear fit, using the software Origin^®^ 7 (Origin Lab Corporation). For a better graphs understanding, we introduce the break in *X*axes, from −4 to −6 logarithm values. The dose-response curve of NNGH was used as a reference value for each determination.
