# Supplementary material for: 4-Thiazolidinone Derivatives as MMP Inhibitors in Tissue Damage: Synthesis, Biological Evaluation and Docking Studies
Source: Molecules. 2018 Feb 14;23(2):415. doi: 10.3390/molecules23020415 (PMC6017844; doi:10.3390/molecules23020415)
Supplement: Supplementary file 1 [file molecules-23-00415-s001.pdf]

Supplementary File

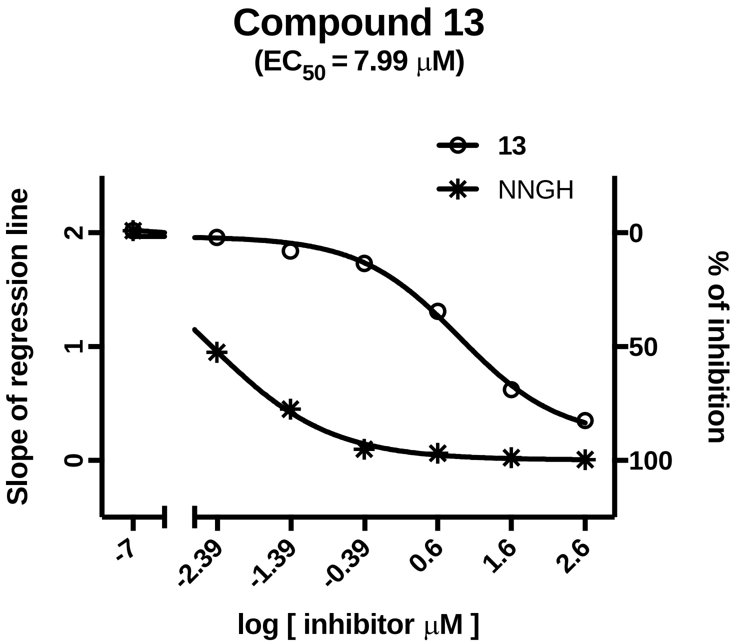

Figure S1. Representative Dose-Response Curve for IC_{50} value determination of compound 13 on MMP-9

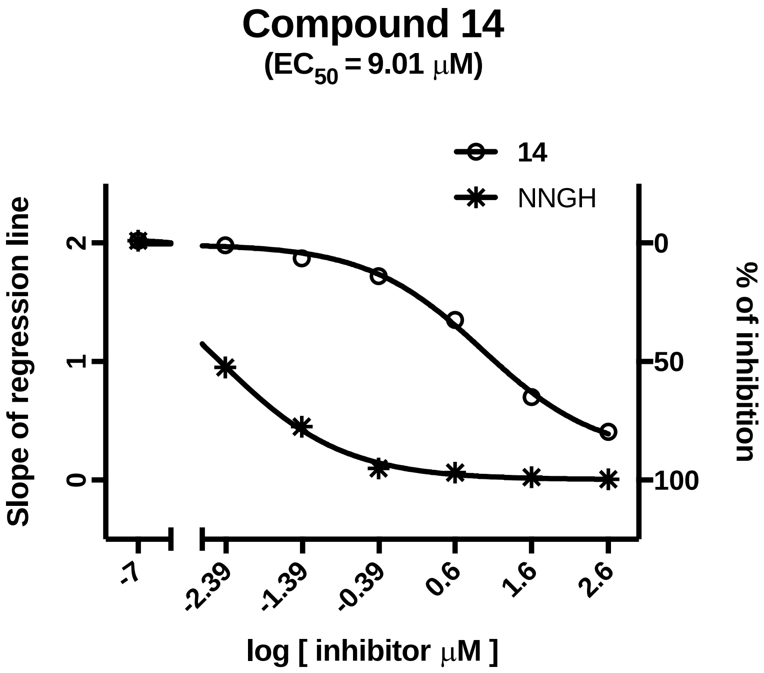

Figure S2. Representative Dose-Response Curve for IC_{50} value determination of compound 14 on MMP-9

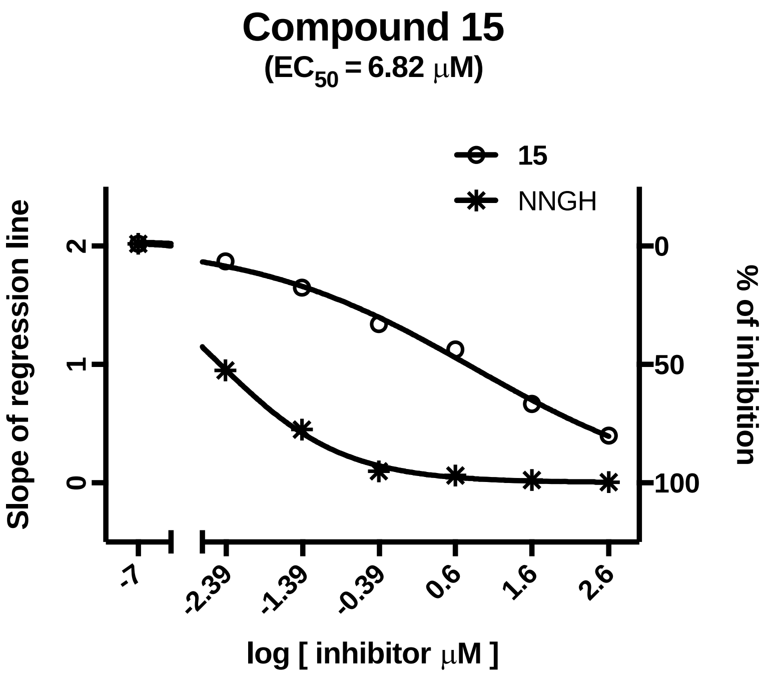

Figure S3. Representative Dose-Response Curve for IC_{50} value determination of compound 15 on MMP-9

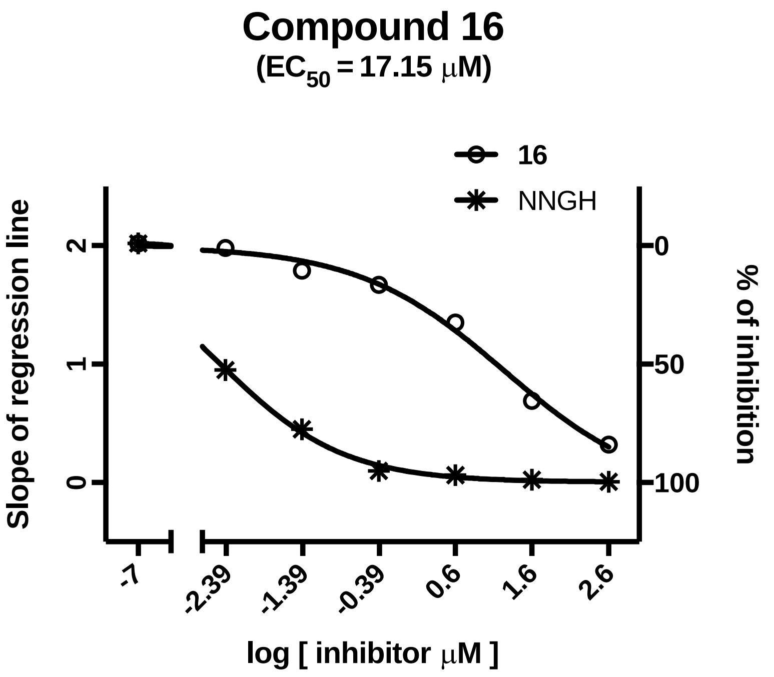

Figure S4. Representative Dose-Response Curve for IC_{50} value determination of compound 16 on MMP-9

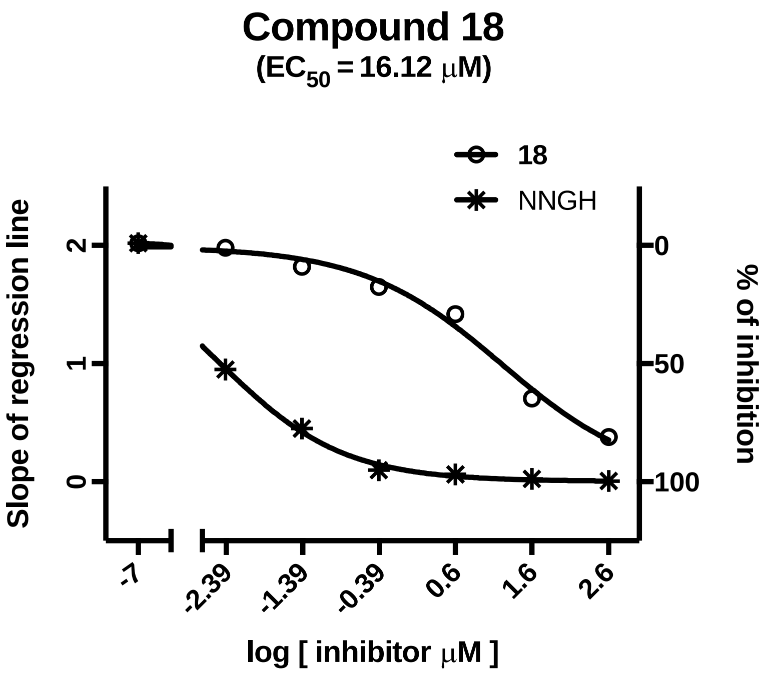

Figure S5. Representative Dose-Response Curve for IC_{50} value determination of compound 18 on MMP-9

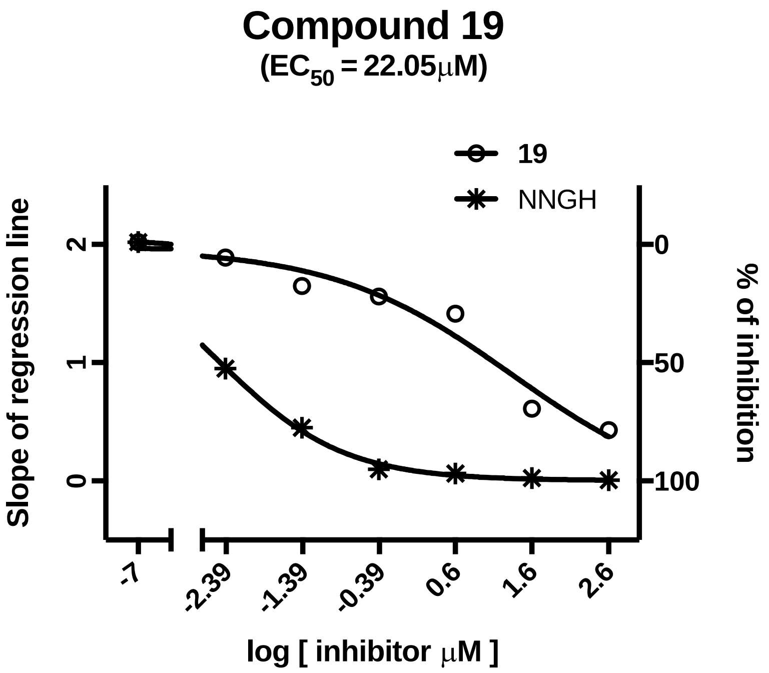

Figure S6. Representative Dose-Response Curve for IC_{50} value determination of compound 19 on MMP-9

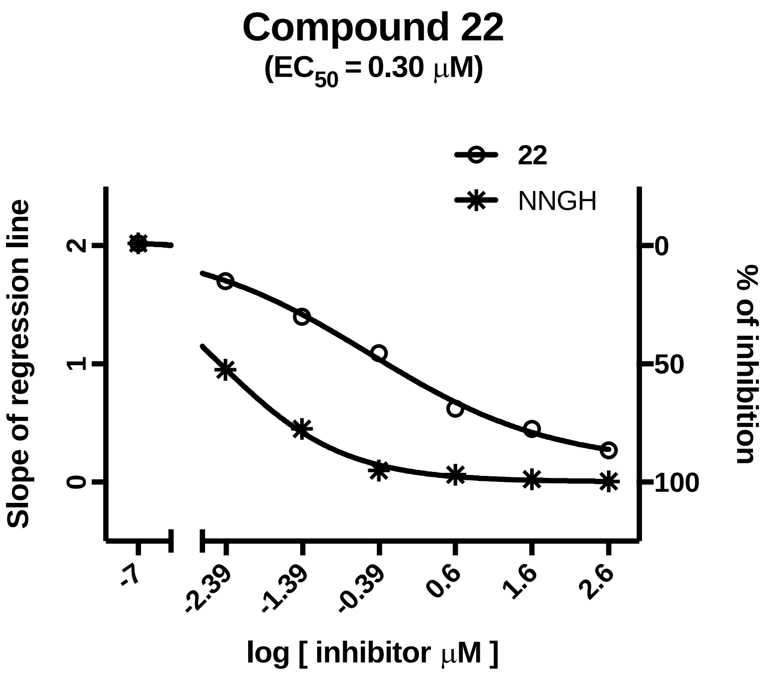

Figure S7. Representative Dose-Response Curve for IC_{50} value determination of compound 22 on MMP-9

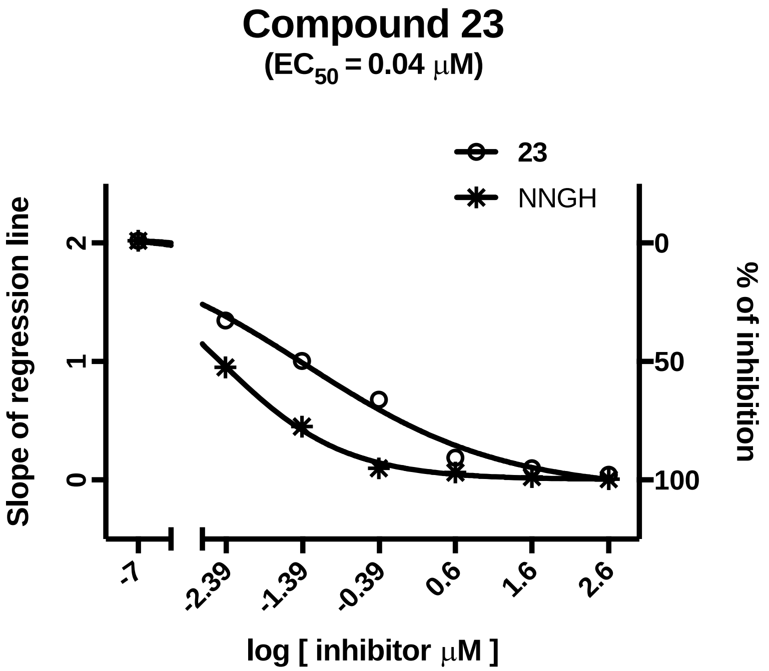

Figure S8. Representative Dose-Response Curve for IC_{50} value determination of compound 23 on MMP-9
